# The accommodative ciliary muscle function is preserved in older humans

**DOI:** 10.1038/srep25551

**Published:** 2016-05-06

**Authors:** Juan Tabernero, Emmanuel Chirre, Lucia Hervella, Pedro Prieto, Pablo Artal

**Affiliations:** 1 Laboratorio de Óptica, Universidad de Murcia, Murcia, Spain

## Abstract

Presbyopia, the loss of the eye’s accommodation capability, affects all humans aged above 45–50 years old. The two main reasons for this to happen are a hardening of the crystalline lens and a reduction of the ciliary muscle functionality with age. While there seems to be at least some partial accommodating functionality of the ciliary muscle at early presbyopic ages, it is not yet clear whether the muscle is still active at more advanced ages. Previous techniques used to visualize the accommodation mechanism of the ciliary muscle are complicated to apply in the older subjects, as they typically require fixation stability during long measurement times and/or to have an ultrasound probe directly in contact with the eye. Instead, we used our own developed method based on high-speed recording of lens wobbling to study the ciliary muscle activity in a small group of pseudophakic subjects (around 80 years old). There was a significant activity of the muscle, clearly able to contract under binocular stimulation of accommodation. This supports a purely lenticular-based theory of presbyopia and it might stimulate the search for new solutions to presbyopia by making use of the remaining contraction force still presented in the aging eye.

Accommodation is a remarkable feature of the visual system allowing to focus object placed at different distances. The crystalline lens changes its shape in a controlled way due to forces applied by the ciliary muscle. The range of distances where the eye can focus properly is reduced with aging, reaching presbyopia (the inability to accommodate) at the end of the 4th decade of live. Both the crystalline lens and the ciliary muscle may have reduced accommodation functionality with age. While it is accepted that the hardening of the lens plays a major role^1^, the functionality of the ciliary muscle seems to be at least partially preserved at presbyopic ages. This is supported by studies where 3D ultrasound bio microscopy (UBM)^2^ and magnetic resonance imaging (MRI)^3^,^4^ were used to monitor the activity of the ciliary muscle during the accommodative process. While both techniques provide useful information to understand the mechanism driving accommodation, they must also face some disadvantages, as UBM is a contact technique (the subject must have an ultrasound prove coupled to the eye) and MRI is limited by image resolution and by a long acquisition time (about several minutes).

In addition to the direct observation of the ciliary muscle, other evidences of the accommodative functionality of the muscle exist^5^,^6^. If a near visual stimulus is presented to the eye, the ciliary muscle would contract releasing tensional forces of the zonular fibers that hold the crystalline lens. Therefore, the lens in the (near) accommodative state would be less attached to the ocular walls than in the (far) unaccommodating form. Any movement of the eye at the same time that accommodation is forced would generate a lens “wobbling” effect theoretically stronger than in far vision conditions^5^. By using this approach, it has been shown that some subjects from a group of presbyopes still had an increase in lens wobbling when accommodation was forced and that could be related to a potentially preserved functionality of the ciliary muscle^6^.

Several studies directly visualizing the ciliary muscle^2^,^3^,^4^, or using indirect techniques^5^,^6^, have reported some functionality of the ciliary muscle at early presbyopic ages (typically 40 to 60 years old). However, it is unknown whether the muscle still operates in older ages in senescence. The reason is that UBM and MRI methods, where contact with the eye and long gaze stability are required, do not seem to be the more optimal techniques to accurately assess the functionality of the ciliary muscle at advanced ages. On the other hand, indirect methods to assess the functionality of the ciliary muscle have used commercially available Dual Purkinje Image eye trackers^7^. It should be noticed, however, that these instruments were primary designed for measuring the direction of gaze. Even with this drawback, researchers estimated how the lens wobbles using data from the overshooting artifact that was typically observed after every saccadic movement of the eye^5^,^6^. The artifact consists of an oscillation of the fourth Purkinje image (rear lens reflection; PIV) with respect to the first Purkinje image (corneal reflection; PI). It occurs typically after every saccadic movement but it cannot be interpreted as a post-saccadic oscillation of gaze but instead as an oscillation of the lens with respect to the cornea (lens wobbling). While this approach permits an indirect quantification of the wobbling phenomenon, it still presents some problems regarding the physical quantification of lens wobbling, like the visualization of the movement (eye trackers only measure and save gaze data) and the extraction of data^8^, the tracking of Purkinje images and the customization of the illumination scheme that generates more clear Purkinje images (see for instance the semi-circular array of infrared LEDs in Tabernero *et al.*^9^).

Recently, an instrument that overcomes those disadvantages has been presented^8^. Based also on the tracking of Purkinje images, this instrument was specifically designed to measure crystalline lens and intraocular lens (IOL) wobbling. The measurement can be quickly performed (a few seconds) and requires no physical contact with the eye. Additionally, the instrument has been specifically modified for this work in order to greatly simplify the subject’s task (they only had to follow a light fixation stimulus under comfortable open-view binocular conditions). By using this new instrument, the objective of this article is to show a clear and robust evidence of a significant functionality of the ciliary muscle at advanced ages well beyond the typical onset of presbyopia.

## Results

### IOL wobbling as the consequence of an inertial decentration of the IOL

**Figure 1:**
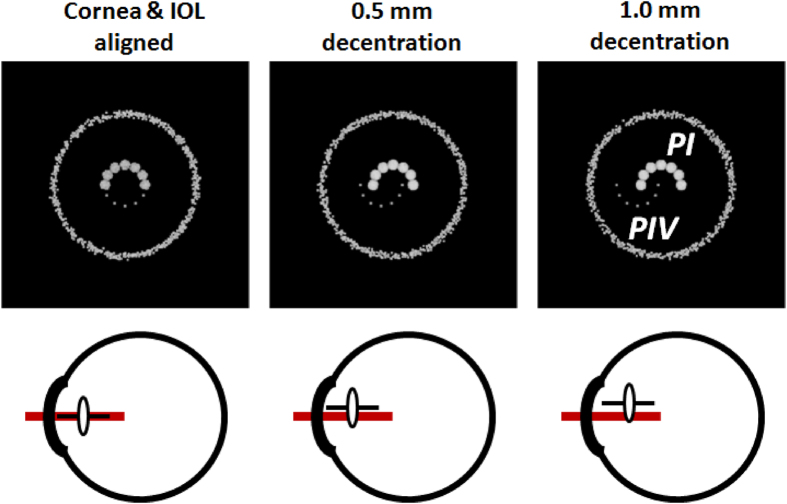
Ray-tracing simulation of Purkinje images for different values of IOL decentration. A semicircular array of point sources emits light towards an eye model implanted with an intraocular lens (IOL). A detector using a tele-objective lens focused on PIV captures the images of the sources reflected from the anterior cornea of the model (PI) and from the posterior surface of the IOL (PIV). The IOL is placed initially co-aligned with the cornea (left panels) and also decentered horizontally by 0.5 and 1.0 mm (middle and left panels). The misalignment of the IOL also generated a shift in the positions of the PIV reflected image along the same direction as the IOL was moved.

A movement of the IOL in the capsular bag after a saccade (i.e. IOL wobbling) is the consequence of the braking deceleration of the eye during the last part of the saccade. It can be experimentally visualized through an image reflected on the IOL (PIV is reflected from the rear IOL surface) compared to an image reflected from the cornea (PI), recorded both at high speed (278 frames per second for this particular application). Here, in order to confirm the effects of the IOL movement on the positions of the lens reflections, we implemented a ray-tracing computer simulation of the realistic eye’s conditions. Assuming that the inertial oscillations of an IOL are essentially generated by a displacement of the lens with respect to the equilibrium position, we used an exact ray-tracing technique to calculate the positions of cornea and lens Purkinje images. The results of the simulation are summarized in Fig. 1. As the amount of IOL decentration increased, the shifts in the positions of the PIV image with respect to PI also increased. In this case, as in the experimental set-up, Purkinje images were generated by a semicircular array of LEDs. This figure described a well-aligned eye (left panels) where both (semicircular) reflections were well aligned and it compares to the situation where a 0.5 mm and 1 mm of IOL decentration were induced (middle and right panels). A significant misalignment between PI and PIV images of up to 1.1 mm for the 1 mm decentration case could be clearly observed from the simulations. We also performed a simulation where the IOL is tilted around the vertical axis. The results shown that to reach the same shifts between PI and PIV as in the 1 mm decentration case, the IOL should be tilted by about 7 degrees.

**Figure 2:**
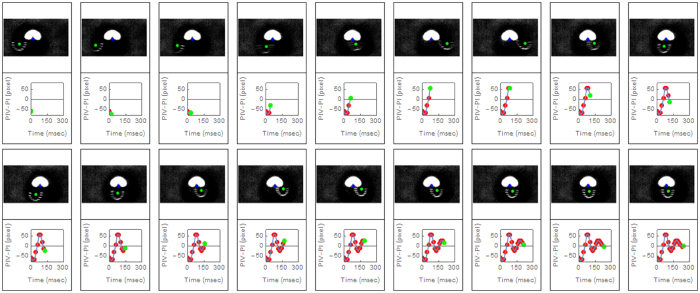
Frame by frame sequence of a 9° saccade movement. The sequence corresponds to subject #1. It begins with the saccadic movement and lasts for 230 msec. Each frame was taken with an exposure time of 3.6 msec and here we only plotted one of every four consecutive frames (temporal resolution is four times higher than represented here). The green dot on the image represents the central position of the semicircular reflex from the posterior IOL (PIV). The graph below each image represents the horizontal distance from the PIV to the PI (corneal reflex) as a function of time. The green dot in each graph corresponded to the current frame on top. A very clear post-saccadic oscillatory behavior of the IOL can be inferred from this sequence.

Similar amounts of Purkinje images shifts where found in the real experiment when the saccadic movement was forced. Figure 2 showed a real sequence of Purkinje images taken in one of the subjects participating in the study. It represented a 250 msec sequence that began with a 9° saccadic movement. The graphs below each image represented the horizontal position of PIV (lens reflection) with respect to the corneal reflection (PI) as a function of time. The oscillating behavior of the lens (IOL in this case) was very clear and evident from the images. A movie clip version of this figure that includes the full temporal resolution range has been included as Supplementary video S1. The magnitude of the maximum PIV-PI shift (1.2 mm) was comparable to the range of shifts obtained in the 1 mm IOL decentered simulation or to the 7 degree IOL tilted simulation. It was possible that decentration or tilt of the IOL dominated alone the wobbling effect, although the combined effect of IOL tilting and decentration could certainly be possible as well.

### IOL wobbling at advanced ages was highly attenuated by pharmacologically induced cycloplegia

The previous section demonstrated that an IOL under inertial braking forces in the eye shows some clear and recordable movement. Anatomically, the capsule of the lens, where the IOL is implanted, is joined to the ciliary muscle by the zonular ligaments. A contraction of the ciliary muscle liberates tension on those ligaments and potentially increases IOL wobbling. For this reason, the relative magnitude of wobbling measured under different conditions on the same subjects can be used to directly test the hypothesis that the ciliary muscle works (i.e. contracts) even at advanced ages. We recorded IOL wobbling under binocular conditions fixating to a close visual stimulus (4D) forcing accommodation as much as possible, and also after paralyzing the ciliary muscle. The muscle contraction was suppressed instilling two drops of Tropicamide 1% in each subject (s#1 was 81 years old, s#2 was 80 years old and s#3 was 68 years old). Each subject waited 20 minutes after administration of the paralyzing drug and the second measurement of IOL wobbling.

**Figure 3:**
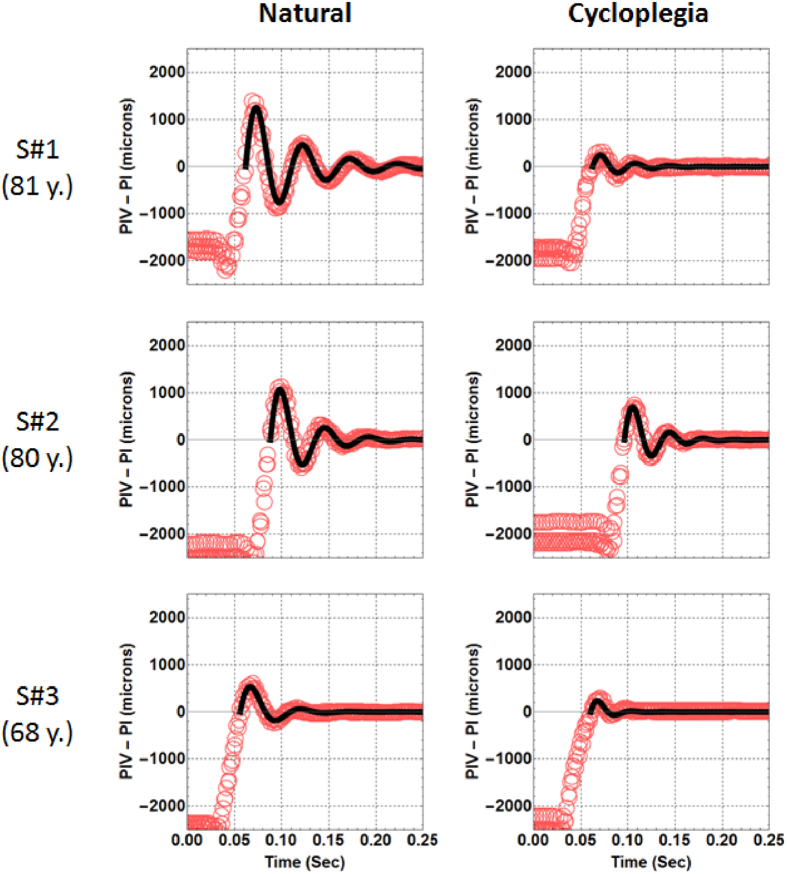
Intraocular lens wobbling data recorded for the three subjects participating in the study. The relative position of PIV with respect to PI is plotted as a function of time under the two conditions of the experiment (natural conditions and 20 minutes after instilling tropicamide in the eye). In both situations, subjects fixated to the same stimulus placed at a distance of 4 D to the eye. Solid lines corresponded to an oscillatory model with exponential decaying amplitude fitted to the experimental data (red open dots).

Figure 3 shows all data collected from the three advanced age subjects in the study, under natural conditions (graphs on the left) and after paralyzing the contraction of the muscle (right). Red open dots represented the distance from the PI to the PIV as a function of time. Data presented in these figures corresponded to three overlapped sequences of saccades. Each saccade sequence was first normalized to zero when it reached the stationary point and after that, all three sets of data were manually overlapped starting all at the beginning point of the saccade. The solid line represents the fitting of the lens wobbling sequence to a harmonic function with an exponentially decaying amplitude. The fitting (a least squares computer routine) was only performed for the data that corresponded to “pure” lens wobbling, i.e. the data from the overshooting behaviour of the oscillation taken with respect to the final stationary location of PIV-PI. Movie clips of the three subjects under both conditions have been included as Supplementary movie clips (Supplementary video S1, Supplementary video S2 and Supplementary video S3 corresponding to subjects S#1 and S#2 and S#3).

**Figure 4:**
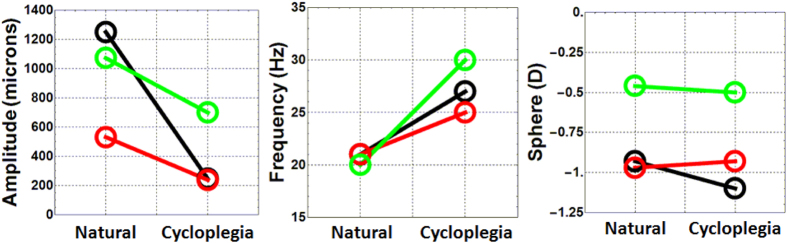
IOL wobbling parameters and control of refractive error. Maximum amplitude of IOL wobbling (left panel), frequency of IOL wobbling (middle panel) and refractive state (right panel) for the three subject participating in the study. Data is presented for the two conditions of the experiment (natural and under pharmacologically induced cyclopegia). The color code represents each subject as in the previous figure (black is s#1; green is s#2; red is s#3).

Figure 3 qualitatively shows that paralyzing muscle contraction had an attenuating affect on IOL wobbling, although there was a significant variety in the magnitude of the effect between subjects and conditions. Figure 4 shows the parameters that quantitatively characterized the wobbling of these three subjects, for both natural and pharmacologically paralyzed accommodation. Left and central panels show the maximum amplitude of IOL wobbling and the oscillation frequency while the panel to the right showed the refraction measured under both conditions. The wobbling amplitude was defined as the maximum value of the first oscillating peak. Under natural conditions, that peak was larger than with the accommodation paralyzed. The decreases in wobbling amplitude varied between subjects (in average was 50%). The oscillation frequency was obtained directly from the fitting of the data to the exponentially decaying harmonic functions. In this case, the measurements with the accommodation paralyzed showed larger frequencies than under natural conditions (in average, a 25% of increment). Objective data on refraction for the far and near targets in each subject during the experiment was obtained with the binocular Hartmann-Shack wavefront sensor. Results were similar in both conditions revealing, as expected, no defocus changes between conditions.

### Saccade dynamics was not affected by pharmacologically induced cycloplegia

**Figure 5:**
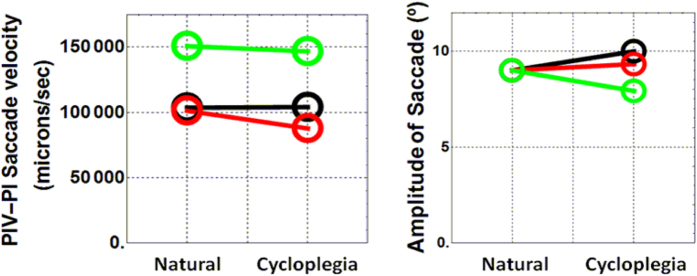
Dynamics of the saccades. Mean saccade velocity (left) and saccade amplitude (right) for the three subject participating in the study. Data is presented for the two conditions of the experiment (natural and under cycloplegia). The color code represents each subject as in previous figures (black is s#1; green is s#2; red is s#3).

IOL wobbling amplitude was clearly attenuated after paralyzing muscle contraction, which was presumably the main reason that validates the initial hypothesis that old subjects still maintain a significant functionality of the muscle contraction. However, an alternative explanation to the attenuated IOL wobbling after instilling tropicamide in the eye could be related to differences in the saccades under natural and pharmacological conditions. This situation was further explored with measurements of the speed and amplitude of the ocular movements (Fig. 5). Mean saccade velocity (Fig. 5, left panel) was obtained from the slope of the linear function that best fitted the PIV-PI data during the ocular movement. Also, the amplitude of the saccades (Fig. 5 right panel) was estimated assuming that all subjects performed the 9-degrees saccade during the measurements under natural conditions. These data were used as a calibration factor to estimate differences to the measurements when drops were instilled. In both cases (velocity and amplitude) the differences between natural and pharmacological conditions were mostly negligible (between 5% to 10% maximum) and could not be used to explain the much stronger differences in the wobbling amplitude and the oscillation frequency (Fig. 4; left and middle panels).

## Discussion

We have shown that paralyzing any remaining sign of accommodation in the pseudophakic subjects participating in this study (ages were 81, 80 and 68 years old respectively) increased the stability of the IOL against saccades when subjects fixated binocularly at very near targets (4 D). The main hypothesis that explains our results was related to the preserved functionality of the ciliary muscle. When subjects fixated binocularly to the near object (a Maltese cross), a strong convergence signal was generated and that was followed by an accommodation attempt, contracting the ciliary muscle as much as possible. This process could release some capsular tension through the zonule and consequently, the capsule and the IOL that remained inside could wobble more in response to ocular movements. On the other hand, if the contraction of the muscle is pharmacologically suppressed, the lens capsule remained fixed to the ciliary muscle under tensional equatorial forces. Therefore, the amplitude of IOL wobbling could be more attenuated than in the natural conditions experiment. This situation intrinsically assumed that a significant part of the accommodation mechanism (ciliary muscle contraction) still worked well at the advanced age of this pseudophakic subjects.

Another evidence supporting the functionality of the muscle came from the observed increased in the oscillation frequencies of the IOLs after paralyzing the contraction of the muscle (Fig. 4 middle panel). In a string analogy, if a system is under a strong tensional force, higher vibrating frequencies are expected. Also in a spring-mass system, the higher the value of the spring constant, the higher the mass oscillation frequency is (in this particular system, the oscillation frequency is directly proportional to the squared root of the spring constant). In our case, the increase in tensional forces in the system could only be attributed to the zonular fibers as a consequence of muscle relaxation.

While the three subjects presented a clear attenuating behavior of the IOL wobbling when tropicamide was used to paralyze the muscle, some differences between subjects existed. Subjects #1 and #3 reached a very similar amount of IOL wobbling attenuation after instilling topicamide although they had very different starting points under natural conditions. Also, the amplitude of wobbling in subject #2 did not decrease under cycloplegia as much as in the other two participants. This variability could be attributed to potential differences in the amount of tropicamide absorbed by subject #2 or even to differences in the temporal response to the drug. On the other hand, the disparity of the IOL wobbling values among subjects under natural conditions could be related to several factors like tensional differences that affect lens capsule after cataract surgery, or differences in the biomechanics of the zonule and even to the design of the particular IOL inserted in the capsule (dimensions, haptics, materials).

The results shown here clearly support a lenticular theory of presbyopia. Since the ciliary muscle was very active in response to a 4 D near stimulus (even for 80 years old subjects), the accommodative loss could be attributed to changes in the lens biomechanics alone and not to an aging effect of the supporting structures of the lens capsule. In this sense, our results, showing a strong functionality of the ciliary muscle at advanced ages, were in agreement with previous works where the ciliary muscle of presbyopes was directly visualized using MRI, USB and anterior chamber OCT^10^,^11^. In summary, our work highlight the importance to optimize future accommodative IOLs designs to work in close concordance with an accommodative remaining force presented in the eye even at advanced ages.

## Methods

### Purkinje-meter combined with an open-view Hartmann-Shack wavefront sensor

Intraocular lens wobbling was characterized in the right eye of three subjects (subject #1 81 years, subject #2 80 years and subject #3 68 years) implanted with monofocal IOLs after forcing 9-degrees saccades. Informed consent was obtained from each subject participating in the study. All the experimental procedures were approved by the University of Murcia ethics committee and were carried out in accordance with the approved guidelines. Subjects had cataract surgery at different times before the experiment (subject #1 5 years earlier; subject #2 15 months earlier; subject #3 5 months earlier). All of them had no complications related to the surgery and a previous ophthalmological analysis confirmed that they all were healthy and IOLs were correctly placed in the capsular bag.

The methodology to measure IOL wobbling was based on recording the oscillations of Purkinje images (front corneal and back surface IOL reflections; PI and PIV respectively) after the subject performs a forced saccade eye movement. In this particular case, video recording was performed at high speed (278 frames per second). An off-line software routine automatically detected PI and PIV locations on the pupil and it allowed the characterization of the relative movement of the IOL with respect to the cornea.

**Figure 6:**
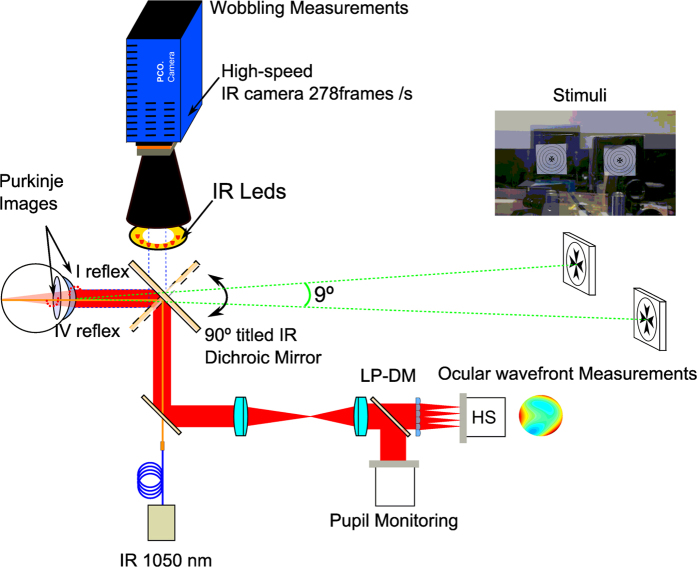
Binocular open-view infrared Hartmann-Shack wavefront sensor combined with an infrared ultra-fast camera. The binocular sensor is used to measure unobtrusively the refraction of both eyes at 1050 nm while the infrared camera records the Purkinje image of a series of IR LEDs at high-speed rate up to 278 frames per seconds. The combination of the HS sensor and the IR ultra-fast camera is possible by means of an infrared dichroic mirror which has the particularity to reflect the infrared wavelength to the HS sensor or to the camera while the visible light is transmitted to the eyes. When the dichroic mirror is rotated to 90° Purkinje images are recorded. In this way the line of sight of the subjects is unchanged and both measurement paths (wobbling and refraction) can be performed quickly and efficiently. The diagram shows, in red, the light path from the retina to the Hartmann-Shack (HS) sensor. The pupil of the subject and the plane of the microlenses are optically conjugated with a telescope. A long pass dichroic mirror (LP-DM; cutting wavelength at 950 nm) divides the light for the HS sensor and for the pupil-monitoring camera.

Additionally, a binocular open-view Hartmann-Shack wavefront sensor^12^,^13^ was used to measure the refractive state and possible change in accommodation of the subjects at the same fixation stimulus used by the wobbling instrument. Both apparatus, the binocular open-view Hartmann-Shack sensor and the Purkinje instrument, were combined together on the same optical table using the same fixation stimulus. A 90° rotation of a hot mirror (both instruments used infrared light to illuminate the eye for the generation of Purkinje images and to measure refraction and aberrations) was used to switch the operating modus of the combined instrument from the wavefront to the wobbling measurements. Figure 6 shows a schematic view of the combined instrument (see legend for details). The fixation stimulus consisted of two Maltese crosses subtending 1 degree each and separated by an angular distance of 9 degrees between centres. Both were placed at a distance of 4 D from the eye. The stimuli were retro-illuminated by white LEDs that flickered with a frequency of 0.5 Hz. The task of the subject was to visualize the illuminated stimulus “as clear as possible”. Because of the special characteristics of this combined optical set-up, all tasks were performed binocularly with an unrestricted field of vision in front of the eyes.

### Ray-tracing computer simulations of Purkinje images

Simulations of the positions of Purkinje images were performed using the non-sequential package of a ray-tracing software (Zemax, Kirkland WA, USA). The geometrical parameters of the eye model were taken from the Liou-Brennan eye model^14^ where the crystalline lens was exchanged by a 21 D IOL made of spherical equi-biconvex surfaces and with 1 mm thickness. Refractive index was 1.458 (λ = 550 nm). The IOL was placed 1 mm away from the pupil plane. A semicircular distribution of point sources was used to simulate the infrared LEDs located on the front aperture of a tele-objective lens. The eye model was placed at 12 cm away from the tele-objective that was focused on the PIV image as in the real experiment. A rectangular detector surface simulated the sensor plane. Then, the first corneal surface and the back surface of the lens were set as perfect reflecting mirror surfaces. Thus, the position of PI (the cornea as a mirror) and PIV (back surface of the lens as a mirror) were simulated in two simple sequential steps.

## Additional Information

**How to cite this article**: Tabernero, J. *et al.* The accommodative ciliary muscle function is preserved in older humans. *Sci. Rep.* **6**, 25551; doi: 10.1038/srep25551 (2016).

## Supplementary Material

Supplementary Information

Supplementary movie 1

Supplementary movie 2

Supplementary movie 3
