# Supplementary material for: The accommodative ciliary muscle function is preserved in older humans
Source: Sci Rep. 2016 May 6;6:25551. doi: 10.1038/srep25551 (PMC4858807; doi:10.1038/srep25551)
Supplement: Supplementary Information [file srep25551-s1.pdf]

TITLE: The accommodative ciliary muscle function is preserved in older humans

Authors: Juan Tabernero[1*], Emmanuel Chirre[1], Lucia Hervella[1], Pedro Prieto[1], and Pablo Artal[1]

[1] Laboratorio de Óptica, Universidad de Murcia, Murcia, Spain;

Supplementary Video Information

Movie clips legends

Supplementary video S1. IOL wobbling under natural accommodated conditions (upper clip) and after paralyzing the activity of the ciliary muscle (down). This sequence corresponds to subject #1 performing a 9 degree saccade under both conditions.

Supplementary video S2. IOL wobbling under natural accommodated conditions (upper clip) and after paralyzing the activity of the ciliary muscle (down). This sequence corresponds to subject #2 performing a 9 degree saccade under both conditions.

Supplementary video S3. IOL wobbling under natural accommodated conditions (upper clip) and after paralyzing the activity of the ciliary muscle (down). This sequence corresponds to subject #3 performing a 9 degree saccade under both conditions.
