# Supplementary figures and images for: The accommodative ciliary muscle function is preserved in older humans
Source: Sci Rep. 2016 May 6;6:25551. doi: 10.1038/srep25551 (PMC4858807; doi:10.1038/srep25551)

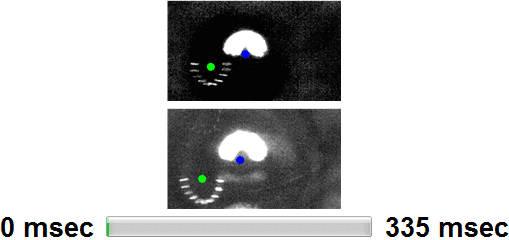

Supplement: Supplementary movie 1 [file srep25551-s2.gif]

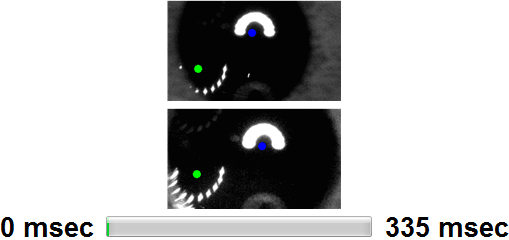

Supplement: Supplementary movie 2 [file srep25551-s3.gif]

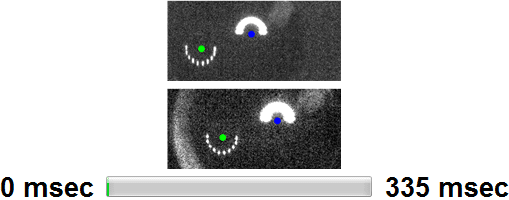

Supplement: Supplementary movie 3 [file srep25551-s4.gif]
